# Screening CZE–UV Green Method for Acetate, Propionate, Butyrate, and Valerate Analysis in Human Stool for Complementary Gut Dysbiosis Diagnosis

**DOI:** 10.1002/elps.70117

**Published:** 2026-05-28

**Authors:** Olívia B. O. Moreira, Matheus Miranda Fracetti, Eliane B. T. Lemos, Jeanette C. F. Less, Lúcio M. Lemos, Paula Rocha Chellini, Marcone A. L. Oliveira

**Affiliations:** ^1^ Grupo de Química Analítica e Quimiometria—GQAQ Chemistry Department Institute of Exact Sciences Federal University of Juiz de Fora (UFJF) Juiz de Fora Minas Gerais Brazil; ^2^ Lemos Laboratório de Análises Clínicas Juiz de Fora Minas Gerais Brazil; ^3^ Faculdade de Farmácia Federal University of Juiz de Fora (UFJF) Juiz de Fora Minas Gerais Brazil; ^4^ National Institute of Science and Technology for Bioanalytics—INCTBio Institute of Chemistry University of Campinas (UNICAMP) Campinas São Paulo Brazil

**Keywords:** capillary electrophoresis, gut dysbiosis, major short‐chain fatty acids, screening method

## Abstract

Short‐chain fatty acids present relevant function in the human body as fermentation metabolites pathways in the natural intestine microbiota activity. Their evaluations can be useful for indication as biomarkers for gut dysbiosis, provided an important health assessment to physicians by specialized laboratories. In the present work, a capillary zone electrophoresis with indirect ultraviolet (CZE–UV) detection method for simultaneous separation of acetate, propionate, butyrate, and valerate in human stool samples was performed. The background electrolyte consisted of 15 mmol L^−1^ of tris(hydroxymethyl)aminomethane (TRIS), 12 mmol L^−1^ of 3,5‐dinitrobenzoic acid (3,5‐DNB) and 0.5 mmol L^−1^ of cetyltrimethylammonium bromide (CTAB) as buffer at pH 7.5, within run time of 7.5 min. The proposed method resulted in an accuracy of 105.5%, 90.12%, 95.9%, and 86.6% and precision of 12.2%, 8.96%, 8.84%, and 18.3% relative standard deviation (RSD) for acetate, propionate, butyrate, and valerate, respectively. The optimized CZE–UV method was successfully applied as potential complementary analytical tool for gut dysbiosis diagnostic assay at 98 stool samples from patients under investigation of intestine‐related anomalies, combining eco‐friendly, quickness, automated, cost‐effective, and high‐throughput aspects. Furthermore, the method offers educational value by exemplifying how sustainable analytical practices can be integrated into modern laboratory training and the teaching of Green Chemistry principles.

## Graphical abstract

A green CZE–UV alternative method for quantifying SCFAs in human stool with cost‐effective and automatization, informative data about patients through reduced sample preparation and minimal consumable. CE is simple and robust equipment for routine clinical laboratory workflows, supporting physicians with information that enhances the accuracy of clinical diagnosis.

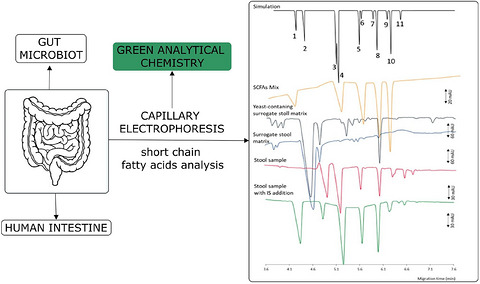

## 1 Introduction

The human microbiota is a complex community composed of non‐pathogenic bacteria and fungi that inhabit the gastrointestinal tract, with considerable interindividual variation in its composition. Gut dysbiosis, being defined as any alteration in the resident microbiota of a healthy individual, is involved in vital metabolic functions, food digestion, and innate and adaptive immunological processes. This condition is an inflammatory, allergenic, and immune‐imbalanced process that may lead to intestinal and extra‐intestinal disorders. Considering an unhealthy lifestyle, inadequate dietary choices, and environmental factors, the human health can be directly affected [1, 2, 3, 4].

Short‐chain fatty acids (SCFAs) are carboxylic acids containing one to six carbon atoms. Microbiota imbalances often arise from the expansion of anaerobic butyrate, producing bacteria belonging to the family Enterobacteriaceae (phylum Proteobacteria), which is considered a common biomarker for dysbiosis [5]. The butyrate, whose the ionic form is butyric acid, is produced in the human body through the bacterial fermentation from undigested carbohydrates such as dietary fiber and resistant starch [6]. In addition to butyrate, acetate and propionate belong to the main group of SCFAs that support metabolic monitoring and reflect gut activity, thereby serving as key biomarkers for dysbiosis and other gut‐related conditions. Among them, acetate is the most abundant (∼60%), followed by propionate (∼25%) and butyrate (∼15%). On the other hand, valerate, in spite of being produced in smaller amounts, provides auxiliary information through the bacterial fermentation of proteins in the gut [7, 8, 9].

**FIGURE 1:**
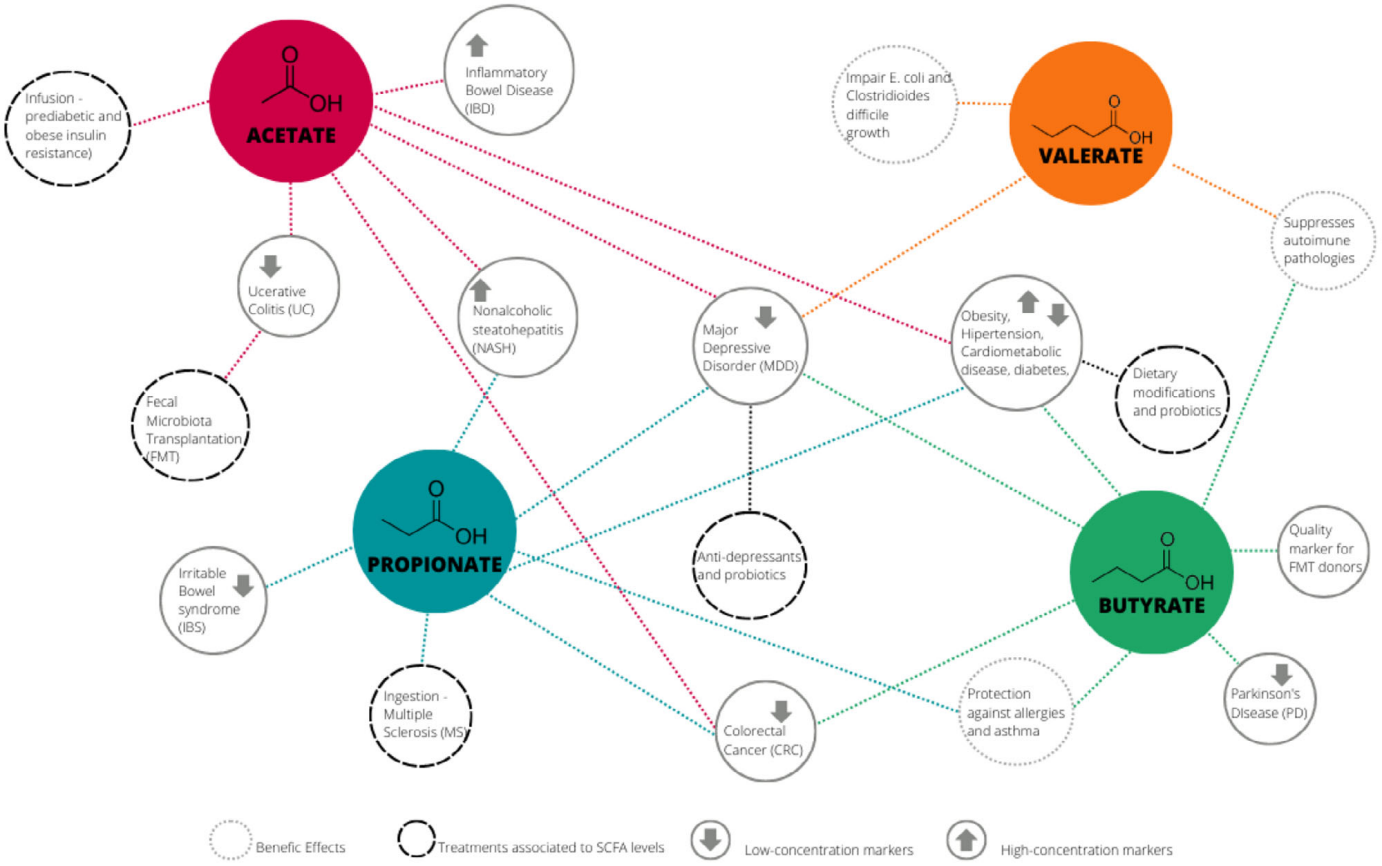
Overview of the potential of SCFAs as health indicators gathered from some research produced in the last 5 years [3, 11, 12, 13, 14, 15, 16, 17, 18, 19, 20, 21, 22, 23, 24, 25, 26, 27, 28].

According to a report by Silva et al., there is growing evidence that SCFAs exert crucial physiological effects on several organs, including the brain [10]. In fact, in recent decades, the functionality of the gut microbiota and the complex relationship between SCFA and human homeostasis have been thoroughly investigated (Figure 1) [3].

Figure 1 summarizes several research topics explored over the past 5 years involving gut dysbiosis in human, as indicated by alterations in SCFA levels. Individually or in combination, each fatty acid may signal the presence of disease and provide insight into its progression. In some cases, therapeutic interventions are guided by modulation of SCFA and are used as markers of beneficial physiological effects that may delay disease onset. Altogether, fecal acetate (C:2), propionate (C:3), butyrate (C:4), and valerate (C:5) quantification have emerged as a valuable auxiliary diagnostic test prescribed by specialists and routinely performed in clinical laboratories to evaluate gut dysbiosis and its associated health imbalances.

SCFAs are usually performed by gas chromatography coupled to mass spectrometry (GC–MS), liquid chromatography with tandem MS (LC–MS/MS), or by enzymatic or volumetric assays. The results are typically expressed as the nominal concentrations of individual or total sums of SCFA. On the other hand, capillary electrophoresis, a separation technique (CE) based on the differential electromigration of analytes according to their intrinsic chemical properties, is an excellent alternative [29]. This technique can be easily implemented and represents an attractive alternative for SCFA quantification, as it enables selective, rapid, eco‐friendly, automated, and cost‐effective analyses compared with conventional methodologies [30]. In this work, they have been described a new approach to evaluating gut‐related metabolic activity based on the simultaneous separation of acetic, propionic, butyric, and valeric acids in human feces using capillary zone electrophoresis with indirect ultraviolet (CZE–UV) with indirect detection and simplified sample preparation protocol.

In addition to the growing demand for sensitive and selective analytical methods, there is an international movement toward the development of techniques aligned with the principles of Green Chemistry [31, 32, 33, 34, 35, 36]. This approach emphasizes the reduction of organic solvent consumption, the minimization of hazardous waste, and the adoption of safer and more sustainable experimental conditions. In this context, CE has emerged as an environmentally favorable alternative to traditional chromatographic techniques, particularly because it operates with extremely small reagent volumes and requires simplified sample preparation steps. Therefore, the selection of the CZE–UV method in this study aligns not only with analytical requirements but also with contemporary sustainability guidelines applied to modern analytical chemistry [37].

## 2 Materials and Methods

### 2.1 Chemicals and Solutions

All solutions used in this study were prepared with water deionized by reverse osmosis system (Quimis, São Paulo, Brazil). Sodium hydroxide (NaOH) was purchased from Synth (São Paulo, Brazil), tris(hydroxymethyl)aminomethane (TRIS) and 3,5‐dinitrobenzoic acid (3,5‐DNB) from Vetec (Rio de Janeiro, Brazil). Cetyltrimethylammonium bromide (CTAB) and all SCFA analytical standards, such as formic (C1:0), acetic (C2:0), propionic (C3:0), butyric (C4:0), and valeric (C5:0), were purchased from Sigma Aldrich (Missouri, EUA). The background electrolyte (BGE) was prepared by mixing aqueous stock solutions of the TRIS/3,5‐DNB buffer and CTAB in freshly deionized water, followed by filtration and storage in amber flask under refrigeration at 4°C until analysis. Aqueous stock solutions of each analytical standard (50 mmol L^−1^) were also stored at 4°C and diluted in freshly deionized water immediately before use.

### 2.2 Samples

Ninety‐eight individuals’ donors, undergo a gastrointestinal investigation, were randomly selected by the Lemos Laboratórios de Análises Clínicas (LLAC). The fecal samples were collected over a 4‐week period. The SCFA analyses were performed using volumetric titration protocol developed by LLAC. The research utilized leftover material from these routine analyses and provided more specific data. Following collection, 1 g of each stool sample was diluted in deionized water (1:10, w/v) and centrifuged at 3500 rpm for 30 min as part of the protein removal protocol that cannot be fully disclosed for commercial reasons. A total of 600 µL of the resulting supernatant was transferred directly to an analytical vial and stored at 4°C. Before analysis, vials were vortexed for 10 s. A pooled sample was used as the analytical quality control (QC) throughout method optimization and validation. All samples were analyzed under a double‐blind protocol in a randomized sequence, interspersed with the calibration curves and QC injections.

### 2.3 Instrumentation and CZE–UV Optimized Method

Experiments were conducted using an Agilent 7100 CE system equipped with an inherent diode array detector (DAD) and controlled by the Agilent OpenLab ChemStation rev C.01.07 (Agilent Technologies, Palo Alto, USA). Samples and standards were injected hydrodynamically into a polyimide‐coated fused‐silica capillary (TSP series; Polymicro Technologies, Phoenix, USA). The aqueous BGE consisted of 15 mmol L^−1^ of TRIS, 12 mmol L^−1^ of 3,5‐DNB (pH ∼ 7.5), and 0.5 mmol L^−1^ of CTAB. In the beginning of the day, the capillary was conditioned by flushing (positive pressure ∼ 950 mbar) with 1 mol L^−1^ NaOH for 40 min, followed by deionized water (20 min) and BGE (20 min). Between runs, the capillary was reconditioned with following sequential flushes (positive pressure ∼ 950 mbar): 1 mol L^−1^ NaOH (60 s), deionized water (30 s), 1 mol L^−1^ HCl (30 s), deionized water rinse (30 s), and BGE (120 s). Additionally, optimized experimental parameters are presented in Table 1.

**TABLE 1:** Optimized parameters of the capillary zone electrophoresis with indirect ultraviolet (CZE–UV) method for simultaneous determination of short‐chain fatty acid (SCFA) in stool samples.

Parameter	Optimized values
Temperature	30°C
Wavelength	240 nm
Capillary effective length	60.0 cm
Total capillary length	68.5 cm
Capillary internal diameter	75 µm
Voltage	−20 kV
Generated current	11 µA
Run time	10 min
Injection program	Sample: 20 mbar, 2 s; IS: 20 mbar, 2 s; BGE (pH ∼ 7.5): 10 mbar, 2 s; wait: 2 s

Abbreviation: BGE, background electrolyte.

### 2.4 Validation and Statistics

The SCAFs were directly quantified using internal standard (IS) calibration on the basis of the ordinary least squares (OLS) method, with C1:0 employed as the IS. When the CE method is considered, the sample matrix complexity associated with the instrumental dispersions, such as sample injection variations and viscosity alteration due to Joule effect and pH change during applied voltage between runs due to electrolysis, becomes the use of IS an important strategy as a boundary condition in order to minimize error. Calibration models were constructed using concentration seven levels for each standard (0.05; 1.65; 3.25; 4.85; 6.45; 8.05; 9.65 mmol L^−1^), whereas the IS concentration was fixed at 5 mmol L^−1^. Three independently prepared calibration curves were analyzed in duplicate in a randomized sequence, interspersed among the sample runs. Linearity was assessed through the correlation coefficient (*r*), lack of fit testing, and regression significance, assuming independence, normality, and homoscedasticity of the data.

The detection and quantification limits of the method, DL and QL, respectively, were calculated using the signal‐to‐noise ratio approach (Equations 1 and 2) on the basis of data obtained from the QC sample:

(1) $$\mathrm{LOD}=\frac{3\times S_{b}\;\times \;C_{x}}{H_{\mathrm{MAX}}-\;H_{\mathrm{MIN}}}$$

(2) $$\mathrm{LOQ}=\frac{10\times S_{b}\;\times \;C_{s}}{H_{\mathrm{MAX}}-\;H_{\mathrm{MIN}}}$$

where $S_{b}$ is the standard deviation of the electropherogram baseline, $C_{x}$ is the concentration of each analyte in the sample, and the denominator corresponds to the height of the respective peak. The factor $S_{b}$ was determined from a randomly selected region of the QC electropherograms using the statistical tools available in Origin software. The value of $C_{x}$ was also calculated from the QC sample according to

(3) $$\;C_{x}=\frac{S_{1}\times \;C_{s\;}\times \;V_{s}}{\left(S_{2-}S_{1}\right)V_{x}}\;$$

where $S_{1}$ is the area of each analyte in the sample, and $S_{2}$ is the area of each analyte in the sample spiked with standard. $C_{s\;}$ is the concentration of the standard solution added to the sample vial, *V* is the volume of solutions added to the vial, being $V_{s}$ and $V_{x}$, volume of standard solution and volume of sample, respectively. These data were acquired from the electropherograms of the QC diluted in water (1:1, v/v), the SCFA standard mix at 5 mmol L^−1^, and the QC diluted in the standard mix (1:1, v/v) resulting in a final concentration of 5 mmol L^−1^ each. The same experiments were used to acquire data for accuracy verification by calculating the recovery percentage, according to

(4) $$R\left(\%\right)=\frac{\left(S_{2-}S_{1}\right)}{S_{3}}\times 100$$

where $S_{3}$ is the area of the analyte in the standard mixture, as $S_{2}$ and $S_{1}$ are the same values abovementioned. All the calculations were performed in triplicates. To complete the method validation, inter‐day and intraday precisions were calculated considering the quantitative analysis of QC placed among the sequence of sample runs. The concentration of each analyte in the QC was determined by using the validated linear models. Ten electropherograms were used for the precision evaluation (five for each day of analysis). All calculations were performed using Microsoft Excel and RStudio, and the results are expressed as a mean value with their respective standard deviations.

All 98 samples were analyzed for the quantitative determination of acetic, propionic, butyric, and valeric acids using the validated calibration models. The complete analytical process, including validation and sample data acquisition, required approximately 72 h. The final results are expressed in mg mL^−1^.

### 2.5 Synthetic Matrix

Synthetic stool samples were used to evaluate matrix effects by adapting a protocol previously optimized by Fabris et al. [38]. A homogenous mixture was prepared using 4 g of polyethylene glycol, 4 g of peanut butter, 1 g of miso paste, 3 g of cellulose, 1 g psyllium husk, 0.5 g calcium chloride, 0.5 g sodium phosphate dibasic, 6 g bakers’ yeast, and 10 mL of freshly deionized water. Because the analytes under investigation originate from fermentative processes, an additional sample was prepared without the baker's yeast for comparison. Following preparation, the synthetic matrix was subjected to the same pretreatment of real samples, as described in Section 2.2.

## 3 Results

From a Green Analytical Chemistry perspective, the analytical protocol was evaluated using GAPI and AGREE tools, which assess compliance with the 12 GAC principles by considering sample preparation, solvents and reagents, instrumentation, and analyst safety. Additionally, the BAGI tool was applied to evaluate the sustainability and efficiency of the method, considering factors such as the number of analytes simultaneously quantified, analysis time, and degree of automation [39, 40, 41].

Table 2 presents the evaluation pictograms for each tool. Overall, the optimized method shows a relatively high degree of “greenness,” with a final AGREE score of 0.73 and a predominance of green pictograms in both the AGREE and GAPI tools. These results may be associated with the use of deionized water as the main solvent, the avoidance of toxic organic solvents, minimal waste generation, and the consumption of only small volumes of BGEs per analysis. Moreover, the absence of derivatization steps and the simplicity of the sample preparation procedure further reduce the environmental impact of the method. In addition, a BAGI score of 77.5 suggests that the analytical method provides a balance between sustainability and analytical performance, allowing the simultaneous quantification of multiple analytes per run, with comparatively short analysis times, and potentially lower energy consumption due to reduced instrumental complexity. In general, these aspects indicate that the proposed CZE–UV protocol may represent a potential alternative to conventional approaches such as GC–MS and LC–MS/MS, depending on the specific application and analytical requirements.

**TABLE 2:** GAPI, AGREE, and BAGI pictograms for the capillary zone electrophoresis with indirect ultraviolet (CZE–UV) optimized method.

GAPI	AGREE	BAGI
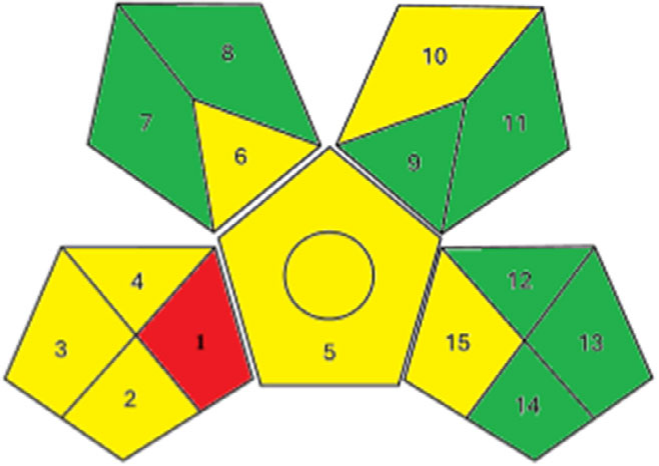	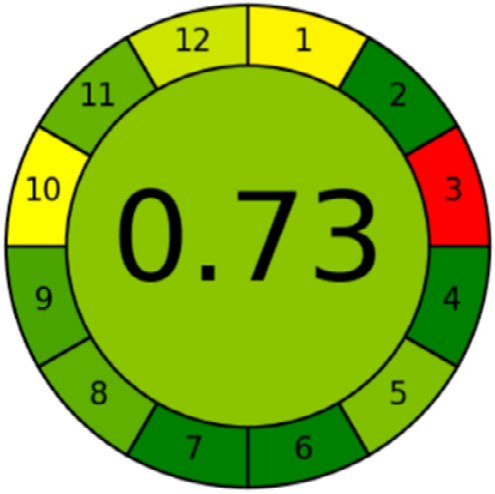	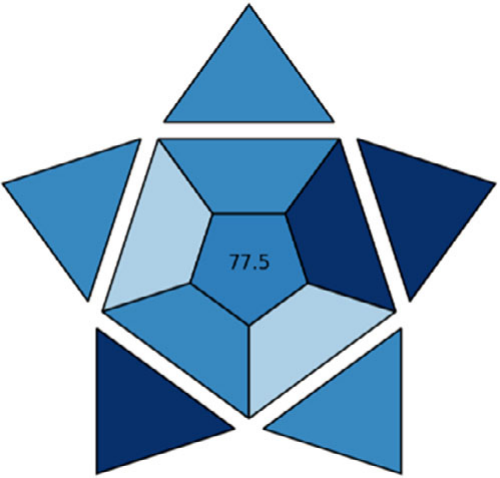

### 3.1 CZE–UV Method Specifics

**FIGURE 2:**
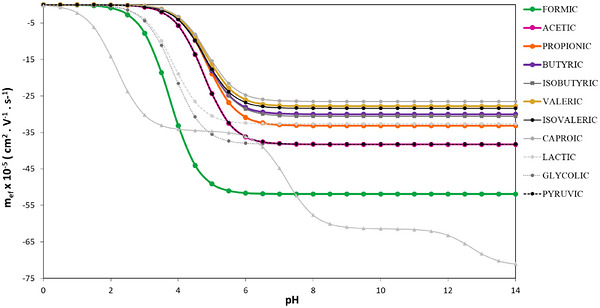
Effective mobility versus pH curves for formic (C1:0), acetic (C2:0), propionic (C3:0), butyric (C4:0), and valeric (C5:0) acids in thicker colored lines and possible interferents in gray‐scale lines. All information concerning the analytes (ionization constants and electrophoretic mobility values) used for the plots was acquired in the PeakMaster simulation software.

The optimized CZE–UV method is based on the separation of five SCFA, C1:0, C2:0, C3:0, C4:0, and C5:0 through their differentiated migration as charged species (ions) in an electric field. As acid–base balance is involved, the analysis is pH‐dependent. The effective mobility versus pH curve of each analyte, described as the product of its intrinsic mobility value (electrophoretic mobility) and the distribution of its concentration at each point of the pH scale [42], provides an overview of the behavior of the species under investigation and their expected separation profile (Figure 2).

**FIGURE 3:**
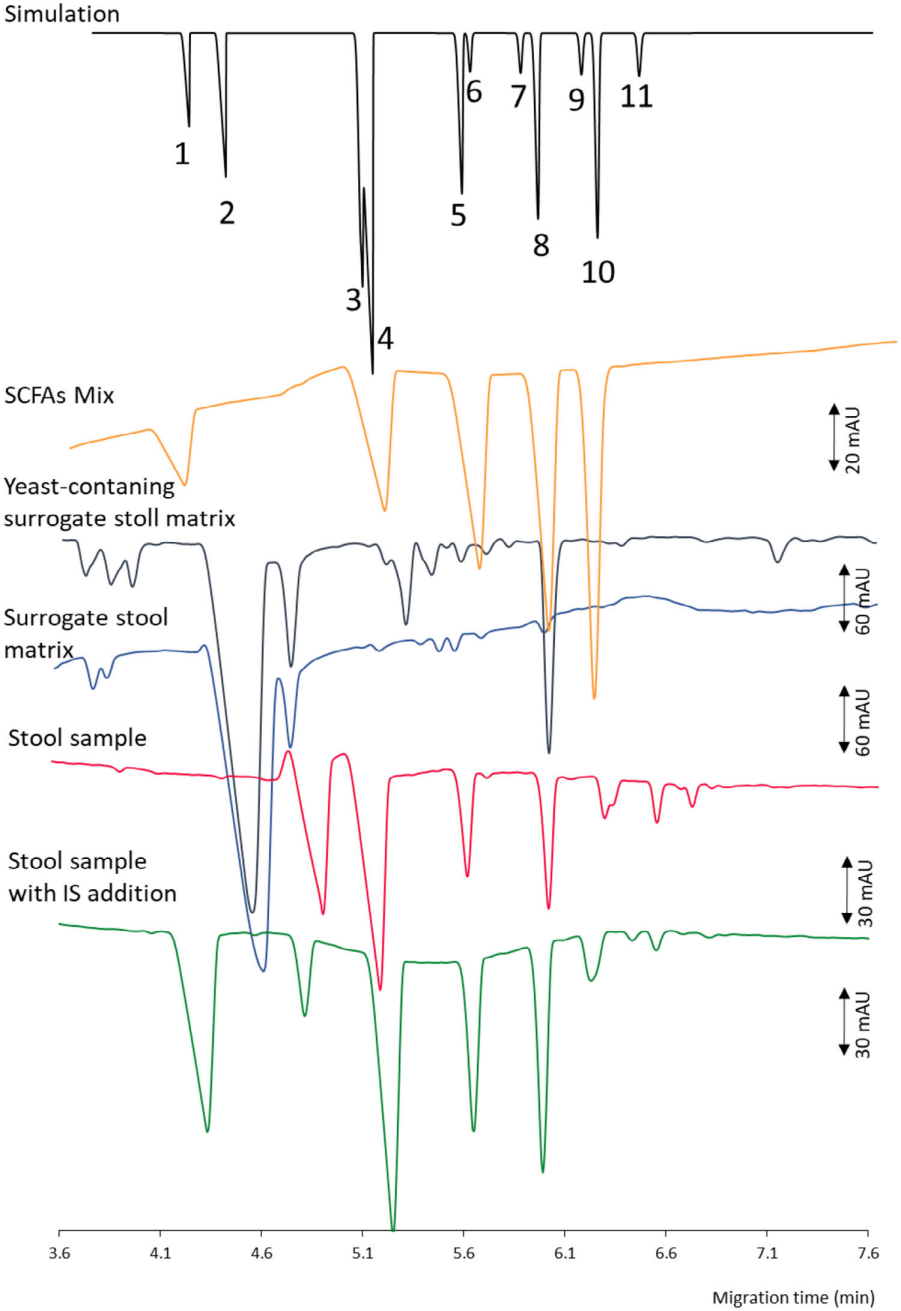
Electropherograms of the PeakMaster simulation containing main analytes and other similar structures: (1) formic acid; (2) phosphates; (3) glycolic and pyruvic acids; (4) acetate; (5) propionate; (6) lactate; (7) isobutyrate; (8) butyrate; (9) isovalerate; (10) valerate; and (11) caproic acid. The standard mixture of the main analytes at 5 mmol L^−1^; surrogate matrices with and without yeast; and two different stool samples with and without on‐column internal standard addition. Experimental conditions described in Table 1. IS, internal standard; SCFAs, short‐chain fatty acids.

The constant values of the effective mobility from pH 6 onward indicate that all species are completely dissociated into their anionic form, thereby being differentiated solely by their charge‐to‐radius ratio. As expected, as the species in focus differ by the incremental addition of a methyl group (–CH_3_) in their structure, in the presence of an electrical field the anions migrate faster as their carbon chain becomes shorter. Additionally, as the SCFAs do not contain chromophore groups in their structure that would allow direct detection by UV–VIS radiation, an indirect detection approach must be considered. In this regard, the BGE ion chromophore (3,5‐DNB) is dislocated when the analytes presenting low molar absorptivity cross in front of the capillary window detection, resulting in signal displacement, which they record as negative peaks in the electropherograms (Figure 3). In fact, it is important to highlight that indirect UV detection presents inherent limitations, such as low sensibility and selectivity, which are very critical when analytes in very low concentration are the investigation aim. Therefore, before to consider UV indirect detection, it is mandatory to check if the concentrations of the analytes in the real samples are compatible with this detection mode. Considering all these aspects, the main electrolyte selected for the experiments was a solution containing an organic acid with a chromophore group in its structure, mixed with a base to form the buffer. 3,5‐DNB/TRIS at pH 7.5 provided an ideal combination, offering good buffer capacity and the chromophore characteristics required for the BGE. When the electrical field is applied in capillary tube filled with BGE, it results in an intrinsic phenomenon characteristic to the internal wall of fused silica capillary column called electroosmotic flow (EOF). Under normal conditions, the EOF is greater than the mobility of anions and moves from the source toward the negatively charged cathode. Consequently, anionic solutes would be pulled back to the source vial by the positively charged anode. To avoid this and enable the analysis, the EOF and the polarity of the applied electric field are inverted [43], allowing the analytes to successfully migrate from the cathode to the anode, which is achieved by adding CTAB to the BGE.

Injection pressure and time, cartridge temperature, capillary length, and voltage were optimized by evaluating the best synergic scenario of good peak resolution, total analysis time, and signal intensity. All optimization experiments were conducted using real stool samples under the different analytical conditions to avoid any possibility of peak coelution that could jeopardize the analysis. The optimized method was applied to samples and to the standard mixture, already considering the on‐column injection of an IS, used for quantitative purposes to minimize matrix effects and improve quantification performance. The on‐column injection of IS provides a better outcome regarding systematic errors and eliminates one step on sample processing [44]. As no traces of formic acid were detected in the samples, it was chosen as the IS for all quantitative analysis (Figure 3).

Overall, regarding method selectivity, bodily fluids are complex matrices in terms of the number of possible interferents; therefore, the analytical method must be selective enough to provide reliable quantitative data. CE is inherently selective, as signal recording can be restricted to a particular molecular group. In this case, the only possible interferents are species with low molar absorptivity, such as organic acids with molecular characteristics similar to those of the main analytes, as well as inorganic anions with similar intrinsic mobility, such as those summarized in Figure 1.

Phosphates, glycolates, pyruvate, lactate, as well as isobutyrate and isovalerate, are metabolites that may be present in feces in small amounts [45, 46, 47]. Comigration with any of the main analytes could compromise the analysis. Based solely on electromobility values (Figure 2), some of these species exhibit similar or even identical migration behavior. However, when considering peak shape, solvation, and the full optimization of the electrophoretic system, the final electropherogram yields a complete separation of all analytes from their potential interferents with good resolution (Figure 3). Real‐life samples exhibit larger peaks than simulated ones, but this does not imply loss of separation; thus, it is not a concern. A resemblance to lactate can be seen in a single stool sample (pink line) when compared with Peaks 5 and 6 of the thinner shaped simulated electropherogram. Similarly, if glycolate and pyruvate (Peak 3) were present in the sample, they would be resolved separately from acetate (Peak 4).

To finalize the experiments regarding method selectivity, the analysis of a feces surrogate matrix was performed. As the main analytes originated from fermentative processes within the human body, two versions of the surrogate matrix, with and without baker's yeast, were prepared for comparison in order to verify conditions where none of the analytes would be detected. Although the matrix electropherogram shows a prominent signal around 4.6 min, in real samples, this unidentified signal can appear only at minimal concentrations or may be metabolized. Nonetheless, even if present, it does not compromise method selectivity. The adjacent signal at approximately 5 min is a matrix component that also does not comigrate with any of the analytes. In the yeast‐containing synthetic sample, the presence of butyrate and a resemblance to acetate are observed, which can be reliably identified by comparison with the standard mixture/simulated data. Overall, we can establish that the method is selective for quantitative evaluations, and any additional perturbations arising from the matrix will be minimized by the use of the IS.

### 3.2 Method Validation

All validation parameters calculated are described in Table 3. DL and QL limits were initially calculated using the signal‐to‐noise approach with data acquired from a stool QC analysis in order to define the lowest point of the analytical working range. On the basis of the reference values expected to be found in human feces, explored in the following sections, the complete linear range was selected. Regarding statistical assumptions, the Shapiro–Wilk normality test and Bartlett's test for homoscedasticity provided *p* values > 0.05 for all SCFAs within 95% confidence interval. Additionally, no lack of fit was observed (*F*_cal_ < *F*_crit_) and all regression significance values (*F*_cal_ is at least 50 times higher than *F*_crit_) [48], along with correlation coefficient close to 1, were acceptable, confirming that the regression models are suitable for quantification purposes. *R* ^2^ = SSReg/SSTot, corresponds to the fraction of the total variability that is explained by the model. However, a model with a high degree of predictability for objects not included in it will present *Q* ^2^ close to 1 and SPRESS close to zero. Therefore, in the present work, all statistical assumptions relating to a good regression model fit were considered adequate. In order to make clear the results obtained, the information about the data used for linear regression models built (Table S1) and the results obtained through the statistical analysis are described in Supporting Information.

**TABLE 3:** Linearity and validation parameters for short‐chain fatty acid (SCFA) determined by the capillary zone electrophoresis with indirect ultraviolet (CZE–UV) optimized method, expressed as a mean value with the respective standard deviation.

SCFA	Acetic	Propionic	Butyric	Valeric
Intercept	0.01673 ± 0.1217	−0.0750 ± 0.1429	−0.1169 ± 0.1499	−0.1527 ± 0.1603
Slope	0.3233 ± 0.0216	0.4035 ± 0.0255	0.4341 ± 0.0268	0.4641 ± 0.0285
*R* ^2^	0.848	0.862	0.867	0.869
*Q* ^2^	0.83	0.85	0.85	0.85
*SPRESS*	0.10	0.14	0.15	0.18
Lack of fit	*F*_cal_ = 0.415 < *F*_crit_ = 2.485	*F*_cal_ = 0.415 < *F*_crit_ = 2.485	*F*_cal_ = 0.671 < *F*_crit_ = 2.485	*F*_cal_ = 0.206 < *F*_crit_ = 2.991
Regression significance	*F*_cal_ = 222.8 > *F*_crit_ = 4.085	*F*_cal_ = 268.6 > *F*_crit_ = 4.085	*F*_cal_ = 261.4 > *F*_crit_ = 4.085	*F*_cal_ = 213.8 > *F*_crit_ = 4.196
Normality	*p* value = 0.0928	*p* value = 0.0642	*p* value = 0.0599	*p* value = 0.0530
Homoscedasticity	*p* value = 0.6931	*p* value = 0.6939	*p* value = 0.6745	*p* value = 0.8789
DL (mg mL^−1^)	0.0157 ± 0.0029	0.0091 ± 0.0017	0.0089 ± 0.0016	0.0441 ± 0.0395
QL (mg mL^−1^)	0.0523 ± 0.0097	0.0327 ± 0.0054	0.0308 ± 0.0055	0.1133 ± 0.1318
Recovery (%)	105.50 ± 17.25_(16.3%)_	90.122 ± 1.519_(16.9%)_	95.93 ± 6.833_(18.5%)_	86.57 ± 5.233_(6.04%)_
Inter‐day precision^a^ (mg mL^−1^)	0.8337 ± 0.1352_(16.2%)_	0.4828 ± 0.0791_(16.4%)_	0.6179 ± 0.0823_(13.3%)_	0.1505 ± 0.0302_(20.0%)_
Intraday precision^b^ (mg mL^−1^)	0.9109 ± 0.1116_(12.2%)_	0.5381 ± 0.0482_(8.96%)_	0.6626 ± 0.0586_(8.84%)_	0.1614 ± 0.0296_(18.3%)_

*Note*: Relative standard deviation (RSD) is expressed in parentheses where needed. Degrees of freedom for (a) lack of fit: *F*_crit_ = 2.485 (*ν*_1_ = 5; *ν*_2_ = 45); *F*_crit_ = 2.991 (*ν*_1_ = 3; *ν*_2_ = 25) and (b) regression significance: *F*_crit_ = 4.085 (*ν*_1_ = 1; *ν*_2_ = 40); *F*_crit_ = 4.196 (*ν*_1_ = 1; *ν*_2_ = 28).

Abbreviations: DL, detection limit; QL, quantification limit.

^a^ Precision: (*n* = 5).

^b^ Precision: (*n* = 10).

Accuracy was evaluated using the recovery percentage approach, based on data obtained from a stool QC sample spiked with a standard mixture. For all SCFA, the recovery values were acceptable, as the relative deviations did not exceed the 20% recommended in the literature [49, 50, 51]. Similarly, the new approaching demonstrated precision in both inter‐day and intraday evaluations, with deviations also not exceeding 20% of the nominal concentration. It is important to highlight that the feces samples present very complex matrix. Each sample analyzed came from individual that was fed different foods, whose gut metabolism can be influenced, including by emotional feelings of the person. Within this context, according to bioanalytical method validation guides [49, 50] and guidelines for calibration in analytical chemistry [51], the limits found in the present work can be considered adequate. Besides, the results demonstrate that adopting an eco‐friendly analytical approach does not compromise precision, sensitivity, or selectivity. This reinforces a central principle of Green Chemistry applied to analytical science: replacing traditional high‐impact methodologies with alternatives that are equally effective, if not superior, while being safer, more economical, and more sustainable.

### 3.3 Analysis of Stool Samples

As previously mentioned, we designed a pilot study to quantify the four main analytes in real human stool samples, presenting a methodological proposal intended for future diagnostic application. Ninety‐seven samples from individuals receiving medical attention for bowel‐related issues were analyzed. The group consisted of 64 women aged 1–91 years and 33 men aged 1–84 years, most of whom were adults over 18 years old. Acetate, propionate, butyrate, and valerate were quantified using the validated model.

Two types of information can be extracted from the resulting dataset. One of them is the total SCFA content, which—when obtained using a separation technique such as CE—corresponds to the sum of the individually quantified main analytes. Additionally, we can assess whether the percentage distribution of each SCFA agrees with the expected the reference values. The combination of these two pieces of information is particularly informative, as even when the total SCFA level falls within the reference range, the relative proportion of each compound may not be within recommended values. Beyond that, because each SCFA plays a distinct role in human homeostasis, evaluating them separately is essential. Therefore, this assay provides an overview of SFCA production as influenced by the patients’ dietary habits and lifestyle.

We emphasize that these results alone should not be used to establish final diagnostic conclusions. Such interpretations must be performed by the physician, considering all relevant clinical information provided by the patients as well as other complementary examinations. Accordingly, our objective here is not to discuss medical outcomes, and no further commentary in that regard is included. From an analytical perspective, the complete dataset provides a broad overview of SCFA levels in the human body, demonstrating that sensitivity is not a limitation and the proposed protocol is comparable to those already disseminated and recognized in the medical community. All samples were also subjected to the gastrointestinal investigational exam COPROMAX by *Lemos Laboratórios*, in which the total SCFA content is determined through a volumetric method. This protocol is based on the titration with a 1 N acidic solution, and the results are expressed as the volume of titrant required to complete the reaction. Therefore, these results are not numerically comparable to those obtained using the proposed CZE–UV protocol. Moreover, titration does not ensure that the quantified substances correspond exclusively to the same analytes detected by the separation method. Overall, on the basis of the results, most cases show indications of bowel dysregulation in both examinations.

To simplify the evaluation of the results, Table 4 presents the range of results obtained with the proposed method, separated by groups, although the reference values remain the same for all. The full dataset containing the results for all 98 samples is provided in Table S2.

**TABLE 4:** Summary of the results of short‐chain fatty acid (SCFA) determined by the optimized capillary zone electrophoresis with indirect ultraviolet (CZE–UV) protocol is expressed as an interval within group of patients.

Group of patients	SCFA total (mg mL^−1^)	Individual SCFA percentage (%)			
		Acetate	Propionate	Butyrate	Valerate
Children	1.28–8.74	22.9–47.5	12.9–40.7	20.3–45.9	2.5–17.3
Adult men	3.01–9.88	8.08–42.8	17.1–33.0	22.8–45.3	6.20–16.9
Adult women	0.68–7.89	5.07–56.7	11.5–40.9	16.0–54.0	2.83–32.3
**Reference values**	**4–18**	**40–75**	**9–29**	**9–37**	**0.5–7**

## 4 Conclusion

CZE–UV is a well‐established and respected CE mode, widely used in academic investigations and capable of offering commercial advantages in the medical field. Our method has proven to be a reliable alternative for quantifying SCFAs in human stool, with cost‐effective and automated benefits. By using this approach, we provide comprehensive and informative data about patients through simple instrumentation, reduced sample preparation, minimal consumable use, and environmentally friendly characteristics, in addition to being easy to operate. Therefore, our goal is to promote the use of simple and robust equipment such as CE in routine clinical laboratory workflows, supporting physicians with information that enhances the accuracy of clinical diagnosis. Beyond its analytical merits, the method developed in this study incorporates several principles of Green Chemistry, such as the significant reduction of reagent consumption, the elimination of hazardous organic solvents, and the simplification of sample preparation steps. These characteristics make the technique not only efficient and cost‐effective but also ecologically responsible, an increasingly important requirement for modern clinical laboratories and for the training of the professionals in analytical and clinical chemistry. The method also holds strong educational potential for courses in analytical chemistry and Green Chemistry, as it clearly illustrates how the rational selection of analytical techniques can reduce environmental impact without compromising data quality. Incorporating CE–UV into teaching activities may help train future professionals to adopt more sustainable laboratory practices and promote the broader dissemination of environmentally responsible analytical methodologies.

## Conflicts of Interest

The authors declare no conflicts of interest.

## Supporting information




**Supporting File**: elps70117‐sup‐0001‐SuppMat.docx.

## Data Availability

The data that support the findings of this study are available in the Supporting Information of this article.
