# Supplementary material for: Screening CZE–UV Green Method for Acetate, Propionate, Butyrate, and Valerate Analysis in Human Stool for Complementary Gut Dysbiosis Diagnosis
Source: Electrophoresis. 2026 May 28;47(8):713–23. doi: 10.1002/elps.70117 (PMC13480509; doi:10.1002/elps.70117)
Supplement: Supplementary file 1 [file ELPS-47--s001.docx]

**Supplementary Material**

**Table 1S**- The independent (x) and dependent(y) variables used to model build for SCFA optimized by CZE-UV

| **Issue** | | **Acetic** | |  | | **Propionic** | |  | | **Butyric** | |  | |  | | **Valeric** | |
| --- | --- | --- | --- | --- | --- | --- | --- | --- | --- | --- | --- | --- | --- | --- | --- | --- | --- |
| ***order*** | ***x*** | | ***y*** | |  | ***x*** | ***y*** | |  | | ***x*** | ***y*** |  | | ***x*** | | ***y*** |
| **1** | 0.050 | | 0.020 | |  | 0.050 | 0.022 | |  | | 0.050 | 0.019 |  | | 0.050 | | 0.022 |
| **2** | 0.050 | | 0.064 | |  | 0.050 | 0.048 | |  | | 0.050 | 0.046 |  | | 0.050 | | 0.038 |
| **3** | 0.050 | | 0.069 | |  | 0.050 | 0.041 | |  | | 0.050 | 0.049 |  | | 0.050 | | 0.050 |
| **4** | 0.050 | | 0.118 | |  | 0.050 | 0.084 | |  | | 0.050 | 0.095 |  | | 0.050 | | 0.060 |
| **5** | 0.050 | | 0.111 | |  | 0.050 | 0.095 | |  | | 0.050 | 0.087 |  | | 0.050 | | 0.056 |
| **6** | 0.050 | | 0.208 | |  | 0.050 | 0.126 | |  | | 0.050 | 0.162 |  | | 0.050 | | 0.116 |
| **7** | 1.660 | | 0.384 | |  | 1.657 | 0.414 | |  | | 1.649 | 0.444 |  | | 1.659 | | 0.481 |
| **8** | 1.660 | | 0.549 | |  | 1.657 | 0.600 | |  | | 1.649 | 0.626 |  | | 1.659 | | 0.653 |
| **9** | 1.660 | | 0.259 | |  | 1.657 | 0.280 | |  | | 1.649 | 0.277 |  | | 1.659 | | 0.290 |
| **10** | 1.660 | | 0.359 | |  | 1.657 | 0.351 | |  | | 1.649 | 0.367 |  | | 1.659 | | 0.377 |
| **11** | 1.660 | | 0.657 | |  | 1.657 | 0.671 | |  | | 1.649 | 0.652 |  | | 1.659 | | 0.670 |
| **12** | 1.660 | | 0.838 | |  | 1.657 | 0.847 | |  | | 1.649 | 0.895 |  | | 1.659 | | 0.904 |
| **13** | 3.269 | | 0.693 | |  | 3.264 | 0.772 | |  | | 3.248 | 0.832 |  | | 3.268 | | 0.889 |
| **14** | 3.269 | | 1.071 | |  | 3.264 | 1.213 | |  | | 3.248 | 1.307 |  | | 3.268 | | 1.353 |
| **15** | 3.269 | | 1.034 | |  | 3.264 | 1.188 | |  | | 3.248 | 1.269 |  | | 3.268 | | 1.314 |
| **16** | 3.269 | | 1.123 | |  | 3.264 | 1.287 | |  | | 3.248 | 1.238 |  | | 3.268 | | 1.245 |
| **17** | 3.269 | | 1.170 | |  | 3.264 | 1.306 | |  | | 3.248 | 1.311 |  | | 3.268 | | 1.486 |
| **18** | 3.269 | | 1.476 | |  | 3.264 | 1.619 | |  | | 3.248 | 1.486 |  | | 3.268 | | 1.787 |
| **19** | 4.893 | | 1.067 | |  | 4.885 | 1.256 | |  | | 4.861 | 1.266 |  | | 4.892 | | 1.379 |
| **20** | 4.893 | | 1.531 | |  | 4.885 | 1.876 | |  | | 4.861 | 1.891 |  | | 4.892 | | 1.962 |
| **21** | 4.893 | | 1.405 | |  | 4.885 | 1.673 | |  | | 4.861 | 1.734 |  | | 4.892 | | 1.772 |
| **22** | 4.893 | | 1.855 | |  | 4.885 | 1.993 | |  | | 4.861 | 2.111 |  | | 4.892 | | 2.174 |
| **23** | 4.893 | | 1.531 | |  | 4.885 | 1.794 | |  | | 4.861 | 1.823 |  | | 4.892 | | 1.820 |
| **24** | 4.893 | | 2.202 | |  | 4.885 | 2.580 | |  | | 4.861 | 2.577 |  | | 4.892 | | 2.742 |
| **25** | 6.538 | | 1.186 | |  | 6.527 | 1.409 | |  | | 6.495 | 1.484 |  | | 6.537 | | 1.620 |
| **26** | 6.538 | | 2.187 | |  | 6.527 | 2.634 | |  | | 6.495 | 2.686 |  | | 6.537 | | 2.758 |
| **27** | 6.538 | | 1.751 | |  | 6.527 | 2.194 | |  | | 6.495 | 2.332 |  | | 6.537 | | 2.430 |
| **28** | 6.538 | | 2.376 | |  | 6.527 | 2.729 | |  | | 6.495 | 2.947 |  | | 6.537 | | 3.059 |
| **29** | 6.538 | | 2.051 | |  | 6.527 | 2.349 | |  | | 6.495 | 2.540 |  | | 6.537 | | 2.635 |
| **30** | 6.538 | | 2.422 | |  | 6.527 | 2.912 | |  | | 6.495 | 3.085 |  | | 6.537 | | 3.133 |
| **31** | 8.047 | | 1.330 | |  | 8.034 | 1.834 | |  | | 7.994 | 1.989 |  | | 8.045 | | 2.095 |
| **32** | 8.047 | | 2.588 | |  | 8.034 | 3.147 | |  | | 7.994 | 3.341 |  | | 8.045 | | 3.409 |
| **33** | 8.047 | | 2.275 | |  | 8.034 | 2.796 | |  | | 7.994 | 2.991 |  | | 8.045 | | 3.140 |
| **34** | 8.047 | | 2.851 | |  | 8.034 | 3.619 | |  | | 7.994 | 3.690 |  | | 8.045 | | 3.709 |
| **35** | 8.047 | | 2.953 | |  | 8.034 | 3.602 | |  | | 7.994 | 3.659 |  | | 8.045 | | 5.186 |
| **36** | 8.047 | | 3.179 | |  | 8.034 | 3.974 | |  | | 7.994 | 4.125 |  | | 8.045 | | 4.121 |
| **37** | 8.727 | | 1.930 | |  | 8.712 | 2.333 | |  | | 8.669 | 2.467 |  | | 8.725 | | 3.217 |
| **38** | 8.727 | | 3.083 | |  | 8.712 | 3.773 | |  | | 8.669 | 3.907 |  | | 8.725 | | 4.032 |
| **39** | 8.727 | | 2.696 | |  | 8.712 | 3.201 | |  | | 8.669 | 3.427 |  | | 8.725 | | 3.569 |
| **40** | 8.727 | | 3.379 | |  | 8.712 | 3.619 | |  | | 8.669 | 4.472 |  | | 8.725 | | 4.568 |
| **41** | 8.727 | | 3.191 | |  | 8.712 | 3.999 | |  | | 8.669 | 4.175 |  | | 8.725 | | 4.412 |
| **42** | 8.727 | | 3.855 | |  | 8.712 | 4.806 | |  | | 8.669 | 5.082 |  | | 8.725 | | 5.231 |

**Table 2S**- The results of SCFA determined by the optimized CZE-UV protocol for all 98 samples.

| ***Samples*** | ***ACETIC*** | ***PROPIONIC*** | ***BUTYRIC*** | ***VALERIC*** | ***TOTAL SCFAs*** | ***% ACETIC*** | ***% PROPIONIC*** | ***%BUTYRIC*** | ***%VALERIC*** |
| --- | --- | --- | --- | --- | --- | --- | --- | --- | --- |
| **1** | 0.06 | 0.15 | 0.37 | 0.22 | 0.81 | 7.37 | 19.01 | 46.07 | 27.56 |
| **2** | 1.22 | 0.43 | 0.98 | 0.35 | 2.98 | 40.97 | 14.47 | 32.83 | 11.73 |
| **3** | 2.74 | 2.89 | 1.34 | 0.28 | 7.25 | 37.84 | 39.81 | 18.45 | 3.90 |
| **4** | 0.98 | 0.42 | 0.46 | 0.20 | 2.06 | 47.52 | 20.62 | 22.30 | 9.57 |
| **5** | 1.50 | 0.61 | 0.80 | 0.27 | 3.18 | 47.19 | 19.13 | 25.30 | 8.38 |
| **6** | 0.76 | 0.54 | 0.44 | 0.13 | 1.86 | 40.59 | 29.19 | 23.40 | 6.81 |
| **7** | 1.46 | 0.69 | 0.52 | 0.17 | 2.84 | 51.43 | 24.27 | 18.17 | 6.13 |
| **8** | 2.10 | 1.10 | 1.30 | 0.21 | 4.71 | 44.58 | 23.34 | 27.52 | 4.56 |
| **9** | 0.95 | 0.59 | 1.21 | 0.43 | 3.19 | 29.85 | 18.66 | 38.10 | 13.39 |
| **10** | 2.37 | 0.94 | 1.05 | 0.30 | 4.67 | 50.81 | 20.22 | 22.49 | 6.49 |
| **11** | 2.42 | 2.16 | 2.85 | 0.36 | 7.80 | 31.00 | 27.76 | 36.60 | 4.63 |
| **12** | 1.65 | 0.27 | 0.44 | 0.17 | 2.53 | 65.39 | 10.70 | 17.33 | 6.57 |
| **13** | 1.93 | 1.07 | 1.56 | 0.37 | 4.93 | 39.20 | 21.72 | 31.57 | 7.52 |
| **14** | 2.27 | 0.81 | 1.12 | 0.27 | 4.45 | 50.86 | 18.12 | 25.04 | 5.98 |
| **15** | 0.29 | 0.71 | 0.49 | 0.16 | 1.65 | 17.41 | 43.34 | 29.65 | 9.60 |
| **16** | 0.29 | 0.25 | 0.34 | 0.10 | 0.99 | 29.69 | 25.06 | 34.87 | 10.38 |
| **17** | 2.42 | 1.06 | 1.65 | 0.44 | 5.56 | 43.45 | 19.07 | 29.59 | 7.88 |
| **18** | 0.73 | 0.53 | 0.46 | 0.13 | 1.84 | 39.55 | 28.59 | 24.96 | 6.90 |
| **19** | 0.87 | 0.51 | 0.76 | 0.23 | 2.37 | 36.73 | 21.49 | 32.00 | 9.78 |
| **20** | 0.89 | 0.48 | 0.54 | 0.17 | 2.08 | 42.95 | 23.30 | 25.79 | 7.96 |
| **21** | 0.56 | 0.36 | 0.33 | 0.12 | 1.37 | 41.26 | 26.38 | 23.92 | 8.44 |
| **22** | 0.97 | 0.42 | 0.38 | 0.16 | 1.93 | 50.39 | 21.63 | 19.47 | 8.51 |
| **23** | 1.03 | 0.60 | 0.80 | 0.27 | 2.71 | 37.96 | 22.33 | 29.73 | 9.98 |
| **24** | 0.53 | 0.35 | 0.40 | 0.08 | 1.36 | 38.80 | 25.99 | 29.36 | 5.85 |
| **25** | 0.98 | 0.57 | 0.43 | 0.08 | 2.06 | 47.47 | 27.58 | 21.06 | 3.89 |
| **26** | 0.11 | 0.19 | 0.30 | 0.11 | 0.71 | 15.31 | 26.64 | 41.93 | 16.11 |
| **27** | 0.94 | 0.45 | 0.48 | 0.15 | 2.02 | 46.46 | 22.29 | 23.90 | 7.35 |
| **28** | 0.68 | 0.33 | 0.34 | 0.07 | 1.42 | 47.91 | 23.48 | 23.79 | 4.82 |
| **29** | 0.74 | 0.25 | 0.27 | 0.09 | 1.35 | 55.18 | 18.34 | 19.74 | 6.74 |
| **30** | 0.83 | 0.28 | 0.52 | 0.14 | 1.78 | 46.57 | 16.01 | 29.28 | 8.13 |
| **31** | 0.91 | 0.50 | 0.67 | 0.24 | 2.32 | 39.14 | 21.42 | 28.98 | 10.46 |
| **32** | 2.36 | 0.95 | 0.87 | 0.12 | 4.30 | 54.82 | 22.07 | 20.24 | 2.87 |
| **33** | 0.80 | 0.38 | 0.35 | 0.21 | 1.74 | 46.06 | 21.94 | 19.90 | 12.10 |
| **34** | 0.53 | 0.39 | 0.47 | 0.15 | 1.54 | 34.68 | 25.42 | 30.32 | 9.58 |
| **35** | 0.53 | 0.36 | 0.44 | 0.16 | 1.48 | 35.66 | 24.19 | 29.67 | 10.48 |
| **36** | 1.45 | 0.66 | 0.79 | 0.21 | 3.10 | 46.59 | 21.12 | 25.51 | 6.78 |
| **37** | 0.63 | 0.56 | 0.65 | 0.24 | 2.08 | 30.17 | 27.10 | 31.40 | 11.33 |
| **38** | 0.35 | 0.26 | 0.34 | 0.09 | 1.05 | 33.59 | 25.00 | 32.42 | 8.99 |
| **39** | 0.46 | 0.29 | 0.29 | 0.13 | 1.17 | 39.39 | 24.48 | 25.17 | 10.95 |
| **40** | 0.36 | 0.27 | 0.42 | 0.16 | 1.21 | 29.66 | 22.44 | 34.90 | 13.00 |
| **41** | 1.08 | 0.71 | 0.53 | 0.19 | 2.50 | 42.99 | 28.33 | 21.27 | 7.41 |
| **42** | 2.09 | 0.73 | 1.07 | 0.38 | 4.26 | 48.97 | 17.09 | 25.12 | 8.82 |
| **43** | 0.83 | 0.52 | 0.39 | 0.13 | 1.87 | 44.46 | 27.90 | 20.84 | 6.80 |
| **44** | 0.79 | 0.44 | 0.49 | 0.10 | 1.81 | 43.35 | 24.35 | 26.95 | 5.35 |
| **45** | 0.65 | 0.39 | 0.47 | 0.06 | 1.57 | 41.43 | 24.85 | 30.13 | 3.60 |
| **46** | 0.70 | 0.35 | 0.48 | 0.19 | 1.72 | 40.76 | 20.37 | 27.94 | 10.92 |
| **47** | 0.29 | 0.22 | 0.22 | 0.10 | 0.83 | 35.11 | 26.12 | 26.97 | 11.80 |
| **48** | 1.31 | 0.78 | 0.76 | 0.15 | 3.00 | 43.59 | 26.12 | 25.30 | 4.98 |
| **49** | 0.48 | 0.29 | 0.39 | 0.10 | 1.26 | 37.89 | 23.19 | 31.20 | 7.72 |
| **50** | 0.45 | 0.37 | 0.55 | 0.08 | 1.45 | 31.16 | 25.75 | 37.75 | 5.33 |
| **51** | 0.58 | 0.27 | 0.45 | 0.10 | 1.40 | 41.24 | 19.42 | 32.15 | 7.19 |
| **52** | 0.65 | 0.49 | 0.80 | 0.12 | 2.05 | 31.68 | 23.64 | 39.01 | 5.67 |
| **53** | 0.37 | 0.34 | 0.37 | 0.16 | 1.25 | 30.00 | 27.14 | 29.94 | 12.91 |
| **54** | 0.66 | 0.49 | 0.28 | 0.09 | 1.52 | 43.29 | 32.20 | 18.68 | 5.83 |
| **55** | 1.17 | 0.35 | 0.46 | 0.11 | 2.09 | 56.03 | 16.68 | 21.80 | 5.49 |
| **56** | 0.09 | 0.16 | 0.22 | 0.07 | 0.54 | 16.99 | 29.22 | 41.22 | 12.57 |
| **57** | 0.07 | 0.20 | 0.26 | 0.08 | 0.61 | 11.07 | 32.97 | 42.29 | 13.66 |
| **58** | -0.01 | 0.13 | 0.25 | 0.09 | 0.47 | -1.60 | 27.81 | 53.60 | 20.19 |
| **59** | 0.53 | 0.36 | 0.37 | 0.11 | 1.37 | 38.71 | 26.42 | 26.73 | 8.15 |
| **60** | 0.98 | 0.35 | 0.45 | 0.11 | 1.89 | 51.74 | 18.32 | 24.07 | 5.87 |
| **61** | 0.70 | 0.31 | 0.80 | 0.09 | 1.90 | 36.99 | 16.27 | 42.08 | 4.66 |
| **62** | 1.03 | 0.68 | 0.50 | 0.21 | 2.42 | 42.35 | 28.04 | 20.78 | 8.83 |
| **63** | 0.66 | 0.38 | 0.41 | 0.13 | 1.58 | 42.00 | 23.90 | 25.83 | 8.28 |
| **64** | 1.71 | 0.81 | 1.15 | 0.29 | 3.95 | 43.17 | 20.58 | 29.04 | 7.22 |
| **65** | 0.26 | 0.26 | 0.27 | 0.09 | 0.87 | 29.21 | 29.88 | 30.44 | 10.47 |
| **66** | 0.47 | 0.39 | 0.47 | 0.26 | 1.59 | 29.87 | 24.37 | 29.61 | 16.15 |
| **67** | 0.61 | 0.34 | 0.27 | 0.12 | 1.34 | 45.57 | 25.38 | 20.39 | 8.66 |
| **68** | 1.47 | 0.91 | 0.92 | 0.07 | 3.37 | 43.53 | 27.07 | 27.39 | 2.01 |
| **69** | 0.86 | 0.60 | 0.55 | 0.16 | 2.18 | 39.64 | 27.46 | 25.36 | 7.54 |
| **70** | 1.38 | 1.04 | 1.36 | 0.33 | 4.11 | 33.51 | 25.38 | 33.12 | 7.99 |
| **71** | 1.51 | 0.63 | 0.33 | 0.17 | 2.64 | 57.25 | 23.81 | 12.64 | 6.30 |
| **72** | 1.18 | 0.45 | 0.32 | 0.16 | 2.11 | 55.88 | 21.43 | 15.27 | 7.42 |
| **73** | 0.48 | 0.35 | 0.46 | 0.14 | 1.43 | 33.20 | 24.66 | 32.11 | 10.04 |
| **74** | 0.68 | 0.48 | 0.41 | 0.15 | 1.71 | 39.73 | 27.90 | 23.79 | 8.58 |
| **75** | 0.62 | 0.36 | 0.38 | 0.06 | 1.42 | 43.60 | 25.57 | 26.71 | 4.13 |
| **76** | 1.38 | 0.63 | 0.53 | 0.24 | 2.78 | 49.76 | 22.53 | 18.93 | 8.79 |
| **77** | 1.97 | 0.73 | 0.99 | 0.30 | 3.99 | 49.30 | 18.26 | 24.89 | 7.54 |
| **78** | 0.94 | 0.43 | 0.44 | 0.14 | 1.95 | 48.29 | 21.96 | 22.78 | 6.97 |
| **79** | 1.89 | 0.53 | 1.59 | 0.12 | 4.13 | 45.77 | 12.90 | 38.45 | 2.88 |
| **80** | 1.03 | 0.51 | 0.31 | 0.09 | 1.95 | 53.11 | 26.28 | 16.17 | 4.45 |
| **81** | 2.53 | 1.00 | 1.43 | 0.35 | 5.30 | 47.64 | 18.90 | 26.88 | 6.58 |
| **82** | 1.22 | 0.64 | 0.96 | 0.28 | 3.11 | 39.27 | 20.67 | 31.00 | 9.06 |
| **83** | 1.74 | 1.21 | 1.00 | 0.31 | 4.26 | 40.85 | 28.42 | 23.43 | 7.29 |
| **84** | 2.15 | 0.98 | 1.02 | 0.07 | 4.22 | 50.89 | 23.21 | 24.15 | 1.75 |
| **85** | 1.54 | 0.90 | 0.98 | 0.37 | 3.79 | 40.68 | 23.73 | 25.92 | 9.67 |
| **86** | 0.49 | 0.36 | 0.40 | 0.14 | 1.39 | 35.49 | 26.08 | 28.61 | 9.83 |
| **87** | 1.19 | 0.50 | 0.70 | 0.26 | 2.66 | 44.93 | 18.89 | 26.25 | 9.93 |
| **88** | 1.21 | 0.66 | 0.83 | 0.24 | 2.95 | 41.08 | 22.42 | 28.23 | 8.27 |
| **89** | 2.63 | 1.25 | 1.87 | 0.39 | 6.14 | 42.84 | 20.33 | 30.46 | 6.37 |
| **90** | 0.90 | 0.62 | 0.62 | 0.20 | 2.35 | 38.35 | 26.49 | 26.54 | 8.62 |
| **91** | 0.78 | 0.47 | 0.51 | 0.14 | 1.90 | 41.23 | 24.58 | 27.04 | 7.14 |
| **92** | 0.92 | 0.64 | 0.47 | 0.12 | 2.15 | 42.73 | 29.58 | 21.98 | 5.72 |
| **93** | 1.16 | 0.63 | 1.20 | 0.23 | 3.23 | 36.03 | 19.58 | 37.31 | 7.08 |
| **94** | 0.85 | 0.39 | 0.37 | 0.20 | 1.80 | 46.97 | 21.60 | 20.50 | 10.94 |
| **95** | 1.26 | 0.52 | 0.84 | 0.40 | 3.02 | 41.76 | 17.14 | 27.77 | 13.33 |
| **96** | 2.73 | 1.40 | 1.78 | 0.48 | 6.40 | 42.72 | 21.95 | 27.87 | 7.46 |
| **97** | 1.29 | 0.42 | 0.68 | 0.13 | 2.51 | 51.38 | 16.64 | 26.92 | 5.06 |
| **98** | 0.63 | 0.60 | 0.43 | 0.20 | 1.86 | 33.85 | 32.18 | 23.34 | 10.63 |

Statistical analysis results obtained for the regression models build for acetic, propionic, butyric and valeric acid optimized by CZE-UV, from output of the *RStudio* script.

**ACETIC**

> summary(mod_Acet)

Call:

lm(formula = y ~ x, data = Acetic)

Residuals:

Min	1Q	Median	3Q	Max

-1.28870 -0.16294 0.03317 0.24562 1.01675

Coefficients:

Estimate Std. Error t value Pr(>|t|)

(Intercept) 0.01673	0.12172	0.137	0.891

x	0.32330	0.02166 14.927	<2e-16 ***

---

Signif. codes: 0 ‘***’ 0.001 ‘**’ 0.01 ‘*’ 0.05 ‘.’ 0.1 ‘ ’ 1

Residual standard error: 0.4236 on 40 degrees of freedom

Multiple R-squared: 0.8478,	Adjusted R-squared: 0.844

F-statistic: 222.8 on 1 and 40 DF, p-value: < 2.2e-16

> pureErrorAnova(mod_Acet)

Analysis of Variance Table

Response: y

Df Sum Sq Mean Sq F value	Pr(>F)

x	1 39.972 39.972 206.5061 3.012e-16 ***

Residuals	40 7.176	0.179

Lack of fit 5 0.401	0.080	0.4147	0.8353

Pure Error 35 6.775	0.194

---

Signif. codes: 0 ‘***’ 0.001 ‘**’ 0.01 ‘*’ 0.05 ‘.’ 0.1 ‘ ’ 1

print(Q2)

[1] 0.8314671

> print(SPRESS)

[1] 0.1018719

**PROPIONIC**

> summary(mod_Prop)

Call:

lm(formula = y ~ x, data = Propionic)

Residuals:

Min	1Q	Median	3Q	Max

-1.33300 -0.22060 0.06927 0.18002 1.36476

Coefficients:

Estimate Std. Error t value Pr(>|t|)

(Intercept) -0.07503	0.14297 -0.525	0.603

x	0.40357	0.02548 15.836	<2e-16 ***

---

Signif. codes: 0 ‘***’ 0.001 ‘**’ 0.01 ‘*’ 0.05 ‘.’ 0.1 ‘ ’ 1

Residual standard error: 0.4975 on 40 degrees of freedom

Multiple R-squared: 0.8624,	Adjusted R-squared: 0.859

F-statistic: 250.8 on 1 and 40 DF, p-value: < 2.2e-16

> pureErrorAnova(mod_Prop)

Analysis of Variance Table

Response: y

Df Sum Sq Mean Sq F value Pr(>F)

x	1 62.074 62.074 231.9985 <2e-16 ***

Residuals	40 9.901	0.248

Lack of fit 5 0.536	0.107	0.4009 0.8449

Pure Error 35 9.365	0.268

---

Signif. codes: 0 ‘***’ 0.001 ‘**’ 0.01 ‘*’ 0.05 ‘.’ 0.1 ‘ ’ 1

> print(Q2)

[1] 0.8473543

> print(SPRESS)

[1] 0.1408554

**BUTYRIC**

> summary(mod_Buty)

Call:

lm(formula = y ~ x, data = Butyric)

Residuals:

Min	1Q	Median	3Q	Max

-1.36479 -0.20774 0.02271 0.25388 1.43456

Coefficients:

Estimate Std. Error t value Pr(>|t|)

(Intercept) -0.11693	0.14991	-0.78	0.44

x	0.43416	0.02685	16.17	<2e-16 ***

---

Signif. codes: 0 ‘***’ 0.001 ‘**’ 0.01 ‘*’ 0.05 ‘.’ 0.1 ‘ ’ 1

Residual standard error: 0.5217 on 40 degrees of freedom

Multiple R-squared: 0.8673,	Adjusted R-squared: 0.864

F-statistic: 261.4 on 1 and 40 DF, p-value: < 2.2e-16

> pureErrorAnova(mod_Buty)

Analysis of Variance Table

Response: y

Df Sum Sq Mean Sq F value Pr(>F)

x	1 71.135 71.135 250.6368 <2e-16 ***

Residuals	40 10.886	0.272

Lack of fit 5 0.952	0.190	0.6709 0.6481

Pure Error 35 9.934	0.284

---

Signif. codes: 0 ‘***’ 0.001 ‘**’ 0.01 ‘*’ 0.05 ‘.’ 0.1 ‘ ’ 1

> print(Q2)

[1] 0.8523203

> print(SPRESS)

[1] 0.155291

**VALERIC**

> summary(mod_Vale)

Call:

lm(formula = y ~ x, data = Valeric)

Residuals:

Min	1Q	Median	3Q	Max

-1.48552 -0.28446 0.04388 0.18851 1.60560

Coefficients:

Estimate Std. Error t value Pr(>|t|)

(Intercept) -0.15273	0.16036 -0.952	0.347

x	0.46407	0.02854 16.259	<2e-16 ***

---

Signif. codes: 0 ‘***’ 0.001 ‘**’ 0.01 ‘*’ 0.05 ‘.’ 0.1 ‘ ’ 1

Residual standard error: 0.558 on 40 degrees of freedom

Multiple R-squared: 0.8686,	Adjusted R-squared: 0.8653

F-statistic: 264.3 on 1 and 40 DF, p-value: < 2.2e-16

> pureErrorAnova(mod_Vale)

Analysis of Variance Table

Response: y

Df Sum Sq Mean Sq F value Pr(>F)

x	1 82.316 82.316 257.4369 <2e-16 ***

Residuals	40 12.456	0.311

Lack of fit 5 1.265	0.253	0.7909 0.5634

Pure Error 35 11.191	0.320

---

Signif. codes: 0 ‘***’ 0.001 ‘**’ 0.01 ‘*’ 0.05 ‘.’ 0.1 ‘ ’ 1

> print(Q2)

[1] 0.8544672

> print(SPRESS)

[1] 0.1768256
